# Opposing Transcriptional Mechanisms Regulate *Toxoplasma* Development

**DOI:** 10.1128/mSphere.00347-16

**Published:** 2017-02-22

**Authors:** Dong-Pyo Hong, Joshua B. Radke, Michael W. White

**Affiliations:** Department of Global Health and Florida Center for Drug Discovery and Innovation, University of South Florida, Tampa, Florida, USA; University of Georgia

**Keywords:** *Toxoplasma gondii*, apicomplexan parasites, development, gene expression, transcription factors

## Abstract

The *Toxoplasma* biology that underlies human chronic infection is developmental conversion of the acute tachyzoite stage into the latent bradyzoite stage. We investigated the roles of two alkaline-stress-induced ApiAP2 transcription factors, AP2IV-3 and AP2IX-9, in bradyzoite development. These factors were expressed in two overlapping waves during bradyzoite development, with AP2IX-9 increasing expression earlier than AP2IV-3, which peaked as AP2IX-9 expression was declining. Disruption of the AP2IX-9 gene enhanced, while deletion of AP2IV-3 gene decreased, tissue cyst formation, demonstrating that these factors have opposite functions in bradyzoite development. Conversely, conditional overexpression of FKBP-modified AP2IX-9 or AP2IV-3 with the small molecule Shield 1 had a reciprocal effect on tissue cyst formation, confirming the conclusions of the knockout experiments. The AP2IX-9 repressor and AP2IV-3 activator tissue cyst phenotypes were borne out in gene expression studies that determined that many of the same bradyzoite genes were regulated in an opposite manner by these transcription factors. A common gene target was the canonical bradyzoite marker BAG1, and mechanistic experiments determined that, like AP2IX-9, AP2IV-3 regulates a BAG1 promoter-luciferase reporter and specifically binds the BAG1 promoter in parasite chromatin. Altogether, these results suggest that the AP2IX-9 transcriptional repressor and the AP2IV-3 transcriptional activator likely compete to control bradyzoite gene expression, which may permit *Toxoplasma* to better adapt to different tissue environments and select a suitable host cell for long-term survival of the dormant tissue cyst.

**IMPORTANCE** *Toxoplasma* infections are lifelong because of the development of the bradyzoite tissue cyst, which is effectively invisible to the immune system. Despite the important clinical consequences of this developmental pathway, the molecular basis of the switch mechanisms that control tissue cyst formation is still poorly understood. Significant changes in gene expression are associated with tissue cyst development, and ApiAP2 transcription factors are an important mechanism regulating this developmental transcriptome. However, the molecular composition of these ApiAP2 complexes and the operating principles of ApiAP2 mechanisms are not well defined. Here we establish that competing ApiAP2 transcriptional mechanisms operate to regulate this clinically important developmental pathway.

## Abstract (precis)

*Toxoplasma* infections are lifelong because of the development of the bradyzoite tissue cyst, which is effectively invisible to the immune system. Despite the important clinical consequences of this developmental pathway, the molecular basis of the switch mechanisms that control tissue cyst formation is still poorly understood. Significant changes in gene expression are associated with tissue cyst development, and ApiAP2 transcription factors are an important mechanism regulating this developmental transcriptome. However, the molecular composition of these ApiAP2 complexes and the operating principles of ApiAP2 mechanisms are not well defined. Here we establish that competing ApiAP2 transcriptional mechanisms operate to regulate this clinically important developmental pathway.

## INTRODUCTION

The molecular switch mechanisms that control bradyzoite development have long been sought but have remained elusive, as have clinical treatments effective against the tissue cyst stage. The life cycle of *Toxoplasma* is heteroxenous, with a sexual reproductive cycle exclusive in the felid host and a second clonal reproductive phase (intermediate life cycle) that is possible in virtually any endothermic animal. There are two competing demands of the *Toxoplasma* intermediate life cycle, (i) expansion of tachyzoite numbers to ensure survival and (ii) production of the bradyzoite-tissue cyst required to transmit the infection to the next host. Our previous studies demonstrated that *Toxoplasma* bradyzoite differentiation is a multistep process requiring slowing of the tachyzoite cell cycle combined with key changes in gene expression and exit from the cell cycle (1). Epigenetic and gene-specific transcription mechanisms are implicated (2), indicating that a transcriptional network regulates these developmental processes. However, the molecular basis of these controls is poorly defined and the *Toxoplasma* factors required are not completely identified.

On the basis of “*in silico*” analysis, Balaji et al. (3) proposed that there is a major lineage of transcription factors in *Apicomplexa* (ApiAP2 factors) that are weakly similar to a group of transcription factors found in plants. The plant-like transcription factors in the *Apicomplexa* harbor one to six Apetala2 (AP2) DNA binding domains (4). The substantial majority of ApiAP2 domains have a single characteristic subtype of the AP2 structural fold. This is determined by a sequence pattern in the DNA binding domain (~60 amino acids) that is strongly similar in the different apicomplexan lineages and has distinct differences from the plant AP2 patterns (5). There is now solid evidence that ApiAP2 factors are *bona fide* transcription factors regulating parasite gene expression through direct binding of specific promoter elements, and many *Apicomplexa* genes transcribed in specific life cycle stages are regulated by this type of mechanism (6–9). For some ApiAP2 factors, the influence on gene expression may be indirect through binding and modifying the activity of other chromatin factors (10). A total of 67 ApiAP2 genes are located in the *Toxoplasma* genome with product names assigned by chromosome location and order (e.g., AP2IV-3 searchable at ToxoDB) (11–13). In other *Apicomplexa* lineages, there are far fewer ApiAP2 genes (e.g., 27 in *Plasmodium falciparum* [3] and 17 in *Cryptosporidium* spp*.* [14]), which may reflect the unique evolution of this protozoan family of parasites (15). Recent comparative genome analysis of lineage-specific apicomplexan species with the free-living relatives *Chromera velia* and *Virella brassicaformis* reveals that a large expansion of AP2-like factors likely occurred prior to the splitting of the chromerids and *Apicomplexa* (15). Thereafter, the ApiAP2 gene family diverged independently in each descending apicomplexan lineage. For example, it is very likely that the smaller number of ApiAP2 factors present in modern *Plasmodium* sp*.* parasites is the result of at least three major reductions in gene content since the *Apicomplexa* split from the chromerids (15). These gene reductions may have had other functional consequences for the evolution of ApiAP2 mechanisms, as so-called master regulators of parasite development equivalent to AP2-G in *P. falciparum* (16) have yet to be identified in *Toxoplasma* or other coccidians, where regulation of developmental gene expression is potentially more complex. Our studies reported here expand our understanding of how ApiAP2 factors regulate the development of the clinically important *Toxoplasma* tissue cyst and establish that there are multiple opposing transcriptional forces competing to preserve asexual replication or shift development toward the formation of the transmissible cyst stage. These ApiAP2 repressors and activators are responsive to stress signals and are active early in tachyzoite-to-bradyzoite differentiation, where they regulate many of the same parasite genes. Our results demonstrate that transcription factors regulating tachyzoite-to-bradyzoite development act at the level of the individual parasite and are not coordinated by the intravacuole environment, which may help explain the stochastic nature of this developmental pathway.

## RESULTS

### AP2IV-3 is one of six ApiAP2 factors expressed early in bradyzoite development.

**FIG 1:**
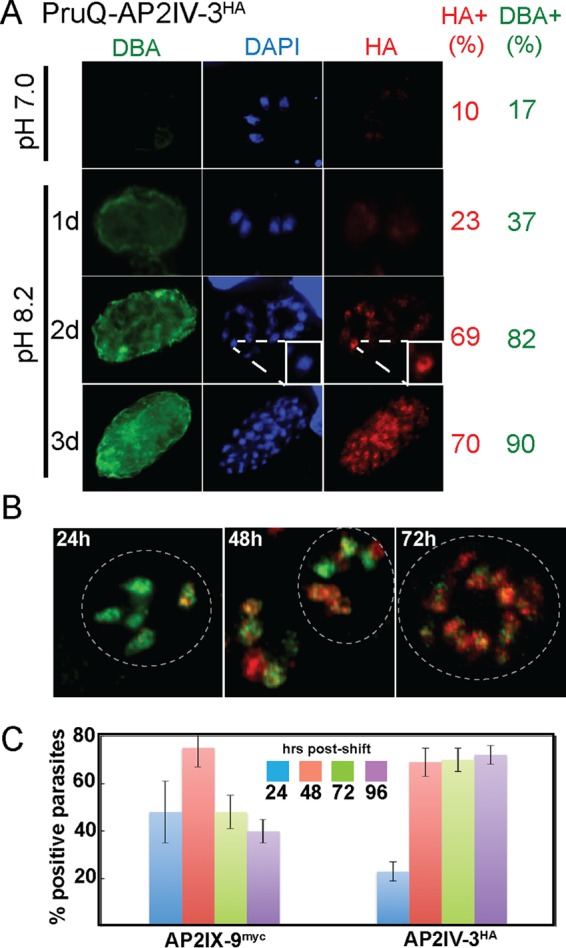
*Toxoplasma* AP2IV-3 is induced by stress during bradyzoite development. (A) The ApiAP2 factor AP2IV-3 was C-terminally epitope tagged by genetic knock-in at the endogenous locus with 3×HA in the PruQ strain. PruQ-AP2IV-3^ha^ parasites were propagated in HFFs on coverslips with pH 7.0 or 8.2 medium. At various times, coverslips were fixed and costained with anti-HA antibody (red, AP2IV-3^HA^ expression), biotin-labeled DBA (green, presence of cyst wall), and DAPI (blue, genomic DNA), followed by the appropriate secondary reagent (see Materials and Methods). The percentages of HA- and DBA-positive parasites in 100 randomly vacuoles were quantified (values on the right). The inset DAPI and anti-HA images of parasites at day 2 after a shift to pH 8.2 medium show colocalization, indicating that AP2IV-3^HA^ was exclusively nuclear. (B, C) Utilizing sequential genetic knock-in at the endogenous loci in the PruQ parent strain, AP2IX-9 (3xmyc) and AP2IV-3 (3×HA) were C-terminally epitope tagged. IFA of the resulting dually tagged transgenic clone was performed with anti-HA (red stain) and anti-myc (green stain) MAbs. Representative vacuoles at 24, 48, and 72 h after an alkaline shift into pH 8.2 medium are shown in panel B. The dashed circles indicate vacuole boundaries. Triplicate quantitative analysis of AP2IX-9^myc^- and AP2IV-3^HA^-positive parasites in 100 randomly selected vacuoles across the 96-h alkaline-shift experiment was performed, and the results are graphed in panel C.

New transcriptome data sets generated from stages across the full *Toxoplasma* life cycle, including the feline sexual cycle, have permitted the reanalysis of ApiAP2 factors (Table 1; see Table S1 in the supplemental material). We selected data sets deposited in ToxoDB (http://toxodb.org/toxo/) representing type II strain transcriptomes from *in vitro* tachyzoites, early and mature bradyzoites, merozoites, and sporozoites to assess how all 67 ApiAP2 mRNAs are developmentally expressed (ranked high to low, 1 to 67, in each sample). For 22 ApiAP2 genes, the mRNA expression data did not identify a specific developmental profile. Many nondevelopmental ApiAP2 factors are expressed at low levels (<50th percentile, 18 Tl), while others showed variable moderate-to-higher expression across the stages. Notably, the cell cycle-regulated tachyzoite factor AP2VI-1 (12) was the only ApiAP2 mRNA expressed at high levels (ranked sixth or higher) across all of the transcriptomes examined (see Table S1). Relatively few ApiAP2 genes were exclusively or highly expressed in a single life cycle stage, whereas multiple life cycle stage expression was more common (Table 1). The expression of 10 ApiAP2 mRNAs was distinctly higher in intermediate life cycles stages (tachyzoites and bradyzoites); however, many more ApiAP2 factors were expressed in these stages plus oocyst environmental stages (10 Tl) or also feline cycle stages (12 Tl) (Table 1). Similar ApiAP2 expression profiles in tachyzoites and bradyzoites were expected because the transcriptomes of these stages are highly correlated (17, 18). Common ApiAP2 expression between the intermediate life cycle and oocyst stages was also anticipated, as more than 20 years ago we established the principle that coccidian sporozoites are preprogramed for the next stage of development (19), which in *Toxoplasma* is the tachyzoite. The expression of ApiAP2 factors during bradyzoite development revealed surprising patterns. A total of six ApiAP2 mRNAs (AP2Ib-1, AP2IV-3, AP2VI-3, AP2VIIa-1, AP2VIII-4, and AP2IX-9) were strongly upregulated in low-passage-number type II ME49 parasites by 2 days following alkaline stress induction of tachyzoites, and four of these factors (AP2Ib-1, AP2IV-3, AP2VI-3, and AP2IX-9) were downregulated in mature bradyzoites (21-day murine brain tissue cysts), indicating that their role is restricted to the transition of the tachyzoite to the bradyzoite. The expression of three of these ApiAP2 factors during early bradyzoite development has been confirmed at the protein level (AP2IV-3, Fig. 1A; AP2Ib-1, not shown; AP2IX-9, reference 6). In this analysis, no ApiAP2 factor was determined to be exclusively upregulated in mature bradyzoites, where instead the dominant phenotype was the downregulation of 11 ApiAP2 mRNAs (Table 1) expressed at higher levels in tachyzoites and/or early bradyzoites. The samples analyzed here are population snapshots and may miss more transiently regulated ApiAP2 factors such as those that are cell cycle regulated (12) and also sexual stage ApiAP2 factors, as these stages were not specifically sampled. In addition, there are strain-specific differences known to affect ApiAP2 expression in *Toxoplasma* (20) that were evident but not further addressed here.

10.1128/mSphere.00347-16.2 TABLE S1  Developmental expression of 67 ApiAP2 *Toxoplasma* mRNAs in transcriptomes representative of the intermediate and feline life cycle stages. Download TABLE S1, DOCX file, 0.3 MB. Copyright © 2017 Hong et al. 2017 Hong et al. This content is distributed under the terms of the Creative Commons Attribution 4.0 International license.

**TABLE 1:** Categories of developmental ApiAP2 mRNA expression

Specific developmental expression	*Toxoplasma* ApiAP2 names
Only expressed in intermediate cycle stages^a^ (at least 1 intermediate stage)	AP2V-1, AP2VIIa-2, AP2VIIa-5, AP2VIIb-3,^a^ AP2VIII-4, AP2IX-4,^a^ AP2IX-9, AP2XI-2,^a^ AP2XI-4,^a^ AP2XII-8
Subset upregulated in early bradyzoites following stress induction of tachyzoites	AP2Ib-1,^d^ AP2IV-3,^d^ AP2VI-3, AP2VIIa-1, AP2VIII-4, AP2IX-9^d^
Subset downregulated in mature *in vivo* bradyzoites	AP2Ib-1,^e^ AP2IV-3,^e^ AP2IV-4, AP2IV-5, AP2V-1, AP2VI-3,^e^ AP2VIIa-5, AP2VIIa-6, AP2VIII-5, AP2IX-9,^e^ AP2XII-6
Only expressed in intermediate cycle stages plus oocyst environmental stages	AP2III-2, AP2IV-5, AP2VIIa-1, AP2VIIa-2, AP2VIIa-3, AP2VIIa-6, AP2VIIa-8, AP2VIII-3, AP2X-5, AP2X-7
Only expressed in intermediate cycle stages plus feline cycle stages	AP2Ib-1,^e^ AP2VI-3,^e^ AP2VIIa-4, AP2VIIa-9, AP2VIII-5, AP2VIII-6, AP2VIII-7, AP2IX-6, AP2IX-8, AP2X-4, AP2X-9, AP2XII-6
Only expressed in feline cycle stages^b^	AP2III-4, AP2IX-6
Only expressed in oocyst environmental stages^c^	AP2III-3, AP2VI-2, AP2VIIa-7, AP2X-2, AP2XII-3,
Only expressed in feline cycle stages and oocyst environmental stages	AP2IV-1, AP2IX-1, AP2X-10

a Intermediate life cycle stages are tachyzoites and early-through-mature bradyzoites. ApiAP2 proteins expressed in all intermediate stages are listed.

b Days 3, 5, and 7 postinfection of the cat host; see ToxoDB.

c Unsporulated oocysts (zygote) to fully sporulated oocysts containing sporozoites.

d Confirmed at the protein level (see Table S1).

e Also specific for early bradyzoites.

AP2IV-3 is one of the six ApiAP2 factors upregulated in early bradyzoites (Table 1), with AP2IV-3 mRNA significantly increased in comparison to tachyzoite expression in response to 48 h of alkaline stress in type II (71st percentile) and type III (84th percentile) strains (http://toxodb.org/toxo/app/record/gene/TGME49_318610). AP2IV-3 mRNA expression in 21-day bradyzoites from mouse brain tissue (18) is substantially downregulated (Table S1). Epitope tagging of AP2IV-3 in a Prugnauid genetic background (Pru-Δ*ku80*Δ*hxgprt*, designated PruQ) by genetic knock-in revealed a nuclear factor that was induced by alkaline stress during the early stages of bradyzoite switching while largely absent from tachyzoites (Fig. 1A).

To better understand AP2IV-3 protein expression, we generated a PruQ transgenic strain by sequential knock-in that expresses dual epitope-tagged ApiAP2 factors, AP2IV-3^HA^ and AP2IX-9^myc^ (Fig. 1B and C). AP2IX-9 is also one the six early-expressed bradyzoite ApiAP2 factors that are downregulated at the mRNA and protein levels in mature bradyzoites (6). Immunofluorescence analysis (IFA) of the dually tagged strain revealed that AP2IX-9^myc^ expression increased before AP2IV-3^HA^ following the change to pH 8.2 medium, while AP2IV-3^HA^ expression became dominant later in development as AP2IX-9^myc^ expression declined (Fig. 1C). AP2IX-9^myc^ and AP2IV-3^HA^ expression patterns were neither synchronous nor exclusive, and parasites sharing a vacuole could be dual or single expressers of these factors (Fig. 1B). The heterologous expression profiles of AP2IX-9^myc^ and AP2IV-3^HA^ were similar to the expression patterns of other bradyzoite proteins (e.g., BAG1, LDH2, and SRS9) that reflect the characteristic asynchronous nature of bradyzoite development (21).

### Genetic disruption and overexpression of AP2IV-3 or AP2IX-9 demonstrate opposite functional roles in the regulation of tissue cyst formation.

**FIG 2:**
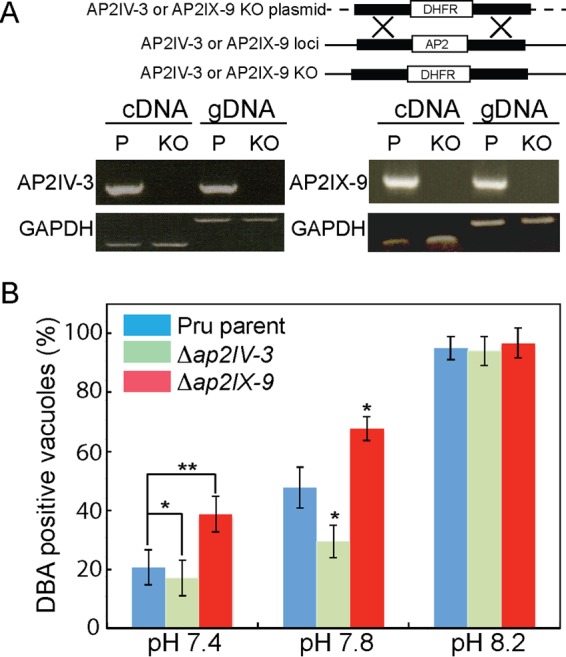
Disruption of the AP2IV-3 and AP2IX-9 genes alters the capacity to form tissue cysts. (A) Schematic representation of knockout constructs for the AP2IV-3 and AP2IX-9 loci. Disruptions of AP2IV-3 and AP2IX-9 were achieved by using the pyrimethamine-resistant dihydrofolate reductase (DHFR)-selectable marker and CRISPR methodology, as shown by the PCR analysis of transgenic DNA (gDNA) and RT-qPCR of cDNA of the parent (P) and knockout (KO) strains. Proper fragment sizes: cDNA, 202 bp; gDNA, 634 bp. PCR analysis of GAPDH was included as a negative control. (B) Tissue cyst formation by the Pru parent strain versus that by Pru-Δ*ap2IV-3* and Pru-Δ*ap2IX-9* knockout parasites grown under different alkaline stress conditions. The proportion of DBA-positive vacuoles in 100 randomly selected vacuoles was assayed in triplicate. Note that disruption of AP2IX-9 (red bars) led to greater tissue cyst formation under milder alkaline conditions, while the opposite result was observed when the AP2IV-3 gene was deleted (green bars). The statistical significance of decreases and increases in cyst formation caused by the loss of AP2IV-3 and AP2IX-9, respectively, is indicated (*, *P* < 0.05; **, *P* < 0.01); green-versus-blue and red-versus-blue statistical comparisons, as diagramed for the pH 7.4 data, were repeated for the pH 7.8 results. No statistically significant difference in cyst formation by Pru parasite strains grown in pH 8.2 medium was observed.

**FIG 3:**
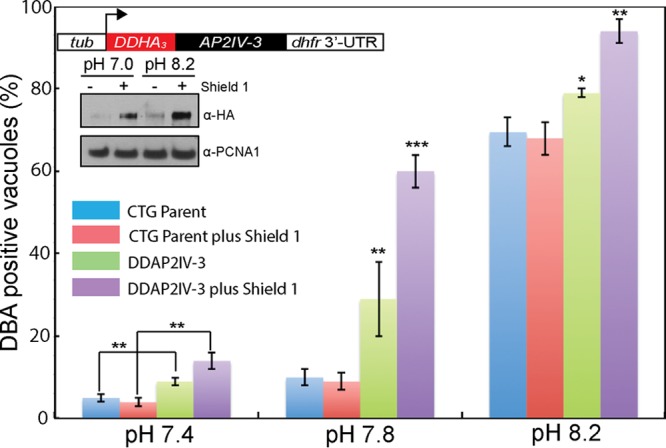
Overexpression of AP2IV-3 enhances tissue cyst formation. The 72-h capacity of the type III CTG parent strain to form tissue cysts compared to that of a CTG transgenic clone expressing ^DD^AP2IV-3 was evaluated after a shift to mild-to-strong alkaline medium plus or minus Shield 1 at 100 nM, as indicated in the color key. The fraction of DBA-positive parasites in 100 randomly selected vacuoles was determined in triplicate. The increases in cyst formation by the ^DD^AP2IV-3 parasites compared to that by the CTG parent control caused by alkaline stress alone or in combination with Shield 1 was statistically significant (*, *P* < 0.05; **, *P* < 0.01; ***, *P* < 0.001; pairwise data comparisons diagramed for the pH 7.4 data were repeated for the pH 7.8 and 8.2 data). Note also that type III CTG parental tachyzoites have a lower propensity to form tissue cysts spontaneously and are more resistant to milder alkaline pH medium conditions than Pru parental strain tachyzoites (Fig. 2B, blue bars). (Inset) Diagram of the ^DD^AP2IV-3 overexpression construct and Western blot assay of ^DD^AP2IV-3 expression with or without Shield 1. Note that ^DD^AP2IV-3 was induced to detectable levels by pH 8.2 medium alone and to higher levels when Shield 1 was added to transgenic parasites grown in pH 7.0 and 8.2 media. The level of nuclear *T. gondii* PCNA1, as revealed by rabbit polyclonal antibody staining, was included as a loading control.

To understand the function of AP2IV-3, we isolated AP2IV-3 knockout clones produced in the Pru strain (Pru = Prugnauid-Δ*hxgprt*) (Fig. 2A) and determined how well Pru-Δ*ap2IV-3* parasites were able to form tissue cysts in alkaline medium (Fig. 2B). We compared these results to those obtained with the Pru parent and also to those obtained with a Pru transgenic clone in which we disrupted the AP2IX-9 gene (Pru-Δ*ap2IX-9*) (Fig. 2A and B). Previous attempts to knock out the AP2IX-9 gene in Pru by conventional methods failed (6), but here, the clustered regularly interspaced short palindromic repeat(s) (CRISPR)-cas9 method was successful (Fig. 1A and Materials and Methods). The Pru parent spontaneously forms 15 to 20% tissue cysts in standard pH 7.0 medium (Fig. 1A) similar to pH 7.4 medium (Fig. 2B, blue bars). Deletion of the AP2IX-9 gene from the Pru strain increased tissue cyst formation to levels higher than those of the Pru parent under mild alkaline stress conditions (Fig. 2B, blue versus red bars, pH 7.4 and 7.8 media). These results are consistent with our previous report that AP2IX-9 is a bradyzoite transcriptional repressor (6). In contrast, deletion of the AP2IV-3 gene resulted in a lower capacity to form tissue cysts in pH 7.4 and 7.8 media (Fig. 2B). Thus, culturing of both transgenic parasites in pH 7.8 medium induced Pru-Δ*ap2IX-9* parasites to form tissue cysts (68%) at more than twice the rate of the Pru-Δ*ap2IV-3* strain in this medium (29%). Complementation of the Pru-Δ*ap2IV-3* strain with a cosmid carrying a single copy of the AP2IV-3 gene fully restored tissue cyst formation to levels that matched or exceeded that of the Pru parent or Pru-Δ*ap2IX-9* parasites (Pru-Δ*ap2IV-3*::AP2IV-3 parasite cyst number results: pH 7.4, 43%; pH 7.8, 72%; pH 8.2, 96%; compare to Fig. 2B). A reduction of tissue cyst capacity was also observed when the AP2IV-3 gene was disrupted in a type III CTG genetic background (Fig. S1, pH 8.2 medium for 72 h). The reduction in tissue cyst formation caused by the loss of AP2IV-3 was more pronounced in the CTG strain than in the Pru strain, which shows higher rates of spontaneous differentiation than the CTG strain and is more readily induced to develop (compare parent blue bars in Pru in Fig. 2B and CTG in Fig. 3). Notwithstanding the strain differences, the results obtained with Pru and CTG transgenic strains demonstrate that AP2IV-3 functions in a manner opposite to that of AP2IX-9 (6) in promoting tissue cyst formation.

10.1128/mSphere.00347-16.1 FIG S1  Disruption of AP2IV-3 by genetic knockout in the type III CTG strain also shows a decrease in the formation of tissue cysts. IFA quantification in triplicate of tissue cyst numbers (DBA positive) formed by CTG parent parasites versus CTG-Δ*ap2IV-3* clones grown in pH 8.2 medium for 72 h. The statistical significance of differences in tissue cyst formation between parent and knockout parasites is indicated (**, *P* < 0.01). Download FIG S1, TIF file, 0.2 MB. Copyright © 2017 Hong et al. 2017 Hong et al. This content is distributed under the terms of the Creative Commons Attribution 4.0 International license.

To confirm the role of AP2IV-3 in promoting tissue cyst formation, we constructed an AP2IV-3 overexpression strain by using the FKBP (DD)/Shield 1 conditional expression model (22–24). The FKBP peptide combined with three copies of the hemagglutinin (HA) epitope tag was fused to the N terminus of the AP2IV-3 coding region (Fig. 3, inset diagram) and then transfected into the type III CTG strain, which has a lower rate of spontaneous differentiation (Fig. 3, blue bars). A clonal CTG-^DD^AP2IV-3 isolate was exposed to increasing alkaline stress plus or minus Shield 1 (100 nM), and the tissue cyst number was determined after 72 h (Fig. 3). As expected, Shield 1-induced overexpression of AP2IV-3 had the opposite effect on tissue cyst formation compared to the loss of AP2IV-3 by genetic knockout. Under mild alkaline medium conditions (pH 7.4 and 7.8 media), tissue cyst formation of the CTG-^DD^AP2IV-3 strain was substantially increased in a Shield 1-dependent manner (Fig. 3). Combination of stronger stress conditions (pH 8.2) with Shield 1 led to nearly 100% of CTG-^DD^AP2IV-3 parasites contained within *Dolichos biflorus* agglutinin-positive (DBA^+^) tissue cysts similar to the images shown in Fig. 1A. This is one of the highest tissue cyst conversion rates that we have observed in any strain *in vitro*. In contrast, previous experiments where AP2IX-9 was overexpressed clearly demonstrated a strong repressive effect on tissue cyst formation in type II and III strains (6).

### AP2IV-3 is an activator of bradyzoite gene expression.

**FIG 4:**
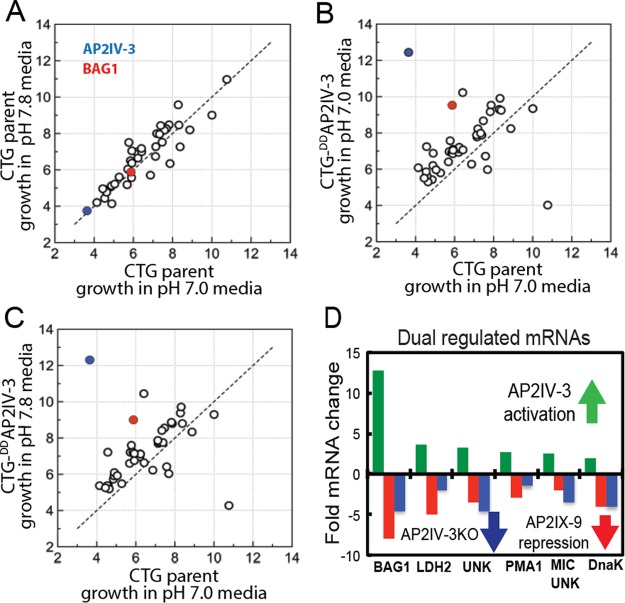
AP2IV-3 is an activator of bradyzoite gene expression. A total of 42 out of 320 alkaline-stress-responsive mRNAs (25), including known bradyzoite genes (see Data Set S1), were affected by Shield 1-induced overexpression of ^DD^AP2IV-3. Comparison of mRNA levels (*x* and *y* axes, log_2_ robust multiarray average values) in CTG-^DD^AP2IV-3 versus CTG parental tachyzoites grown in pH 7.8 or 7.0 medium, all plus 100 nM Shield 1. (A) The expression of the 42 mRNAs in CTG parental tachyzoites grown in pH 7.8 medium versus pH 7.0 medium was unchanged; 98% of the 320 alkaline-stress-responsive mRNAs are <2-fold different (see Data Set S1). (B) Comparison of CTG-^DD^AP2IV-3 tachyzoites grown in pH 7.0 medium versus the CTG parent in pH 7.0 medium revealed that Shield 1-induced overexpression of AP2IV-3 altered 42 alkaline-stress-responsive mRNAs >1.5-fold up or down. The Shield 1-induced overexpression of AP2IV-3 (blue dot) and upregulation of Bag1 mRNA levels (red dot) are indicated. (C) No further changes in mRNA levels over what is shown in panel B were observed when CTG-^DD^AP2IV-3 parasites were grown in mildly alkaline medium (pH 7.8). This indicated that overexpression of AP2IV-3 had a greater influence than mild alkaline stress in altering the expression of the 42 mRNAs. (D) Some mRNAs upregulated by the overexpression of ^DD^AP2IV-3 with Shield 1 (green bars) are repressed when ^DD^AP2IX-9 is also overexpressed (red bars), and these same genes are no longer alkaline stress inducible in parasites lacking AP2IV-3 (blue bars). ^DD^AP2IX-9 fold changes were published previously (6) and are included here as a reference. The data for the seven selected mRNAs shown were derived from the following experiments: ^DD^AP2IV-3 CTG parasites grown in pH 7.0 medium plus Shield 1 compared to the parent strain (green bars and arrow), ^DD^AP2IX-9 in parasites cultured in pH 8.2 medium plus Shield 1 (6) (red bars and arrow), and Pru-Δ*ap2IV-3* parasites grown in pH 8.2 medium without Shield 1 (blue bars and arrow). Gene IDs: BAG1, TGME49_259020; LDH2, TGME49_291040; UNK, unknown gene TGME49_207210; PMA1, TGME49_252640; MIC UNK, TGME49_208740; DnaK, TGME49_202020.

To identify the genes potentially controlled by AP2IV-3, duplicate RNA samples were prepared from CTG parent and CTG-^DD^AP2IV-3 and CTG-Δ*ap2IV-3* transgenic strains grown under different medium conditions plus 100 nM Shield 1 (Fig. 4; see Data Set S1). Changes in mRNA expression in ^DD^AP2IV-3 parasites grown in pH 7.0 medium plus Shield 1 revealed that 42 of the previously published alkaline-stress-responsive genes (a total of 320) (25) were affected compared to CTG parental tachyzoites (compare Fig. 4A and B). A total of 36 genes were upregulated, while only 6 were downregulated (see Data Set S1). Many of the genes upregulated by AP2IV-3 overexpression in tachyzoites (pH 7.0 medium plus Shield 1) are known bradyzoite genes that are induced by strong alkaline stress in the parental CTG strain (see Data Set S1), including the canonical bradyzoite markers BAG1 (^DD^AP2IV-3 tachyzoites, up ~12-fold) and LDH2 (up ~4-fold), as well as a bradyzoite-specific rhoptry protein (26), BRP1 (up ~14-fold) and the SAG-related surface antigen SRS22A (up ~4-fold). A single gene encoding an unknown protein containing several ankyrin repeats was the only strongly downregulated mRNA. For these 42 putative AP2IV-3 target genes, a combination of mild alkaline stress conditions (pH 7.8 medium) with Shield 1 (Fig. 4C) gave results equivalent to those obtained with pH 7.0 medium plus Shield 1 (Fig. 4B), indicating that ^DD^AP2IV-3 overexpression was a primary factor regulating these 42 mRNAs. Consistent with this result, disruption of the AP2IV-3 gene largely led to the opposite affect on gene expression. The majority of the 36 genes (24/36) increased by overexpression of ^DD^AP2IV-3 in tachyzoites failed to be induced by strong alkaline stress when AP2IV-3 was absent (Fig. 4D; see Data Set S1). For example, BAG1 mRNA expression in alkali-stressed CTG-Δ*ap2IV-3* parasites reached only ~10% of the level of BAG1 mRNA induced by alkaline stress of CTG parental parasites. Intriguingly, a number of the genes regulated by AP2IV-3 are also controlled by AP2IX-9 (6), including BAG1 and LDH2, although with opposite effects; overexpression of AP2IV-3 increased, whereas elevated AP2IX-9 repressed, mRNA expression (comparison in Fig. 4D). The mRNA encoding AP2IV-3 is also upregulated in merozoites isolated from cats and in mature sporozoites (see Table S1), suggesting that this factor may have multiple roles in the regulation of gene expression across the *Toxoplasma* life cycle (17). Consistent with this idea, some of the genes upregulated by AP2IV-3 overexpression are also increased in merozoites and sporozoites (see Data Set S2). For example, the rhoptry protein BRP1 (26) is elevated 14-fold by AP2IV-3 overexpression in tachyzoites and is ~10-fold higher in merozoites than in tachyzoites of the same strain (see Data Set S2).

10.1128/mSphere.00347-16.3 DATA SET S1  Analysis of 320 previously published alkaline-stress-responsive genes (25) in CTG parental and CTG transgenic strains (CTG-Δ*ap2IV-3* and CTG-^DD^AP2IV-3) grown in pH 7.0 to 8.2 medium as indicated. The complete microarray data for this experiment have been deposited in GEO (GSE89469). Download DATA SET S1, XLSX file, 0.04 MB. Copyright © 2017 Hong et al. 2017 Hong et al. This content is distributed under the terms of the Creative Commons Attribution 4.0 International license.

10.1128/mSphere.00347-16.4 DATA SET S2  Expression profiles of 42 mRNAs altered by AP2IV-3 Shield 1 overexpression in ME49 parasites grown in pH 7.0 and 8.2 media and M4 strain merozoites and sporozoites. Download DATA SET S2, XLSX file, 0.1 MB. Copyright © 2017 Hong et al. 2017 Hong et al. This content is distributed under the terms of the Creative Commons Attribution 4.0 International license.

### AP2IV-3 directly regulates the transcription of the BAG1 gene.

**FIG 5:**
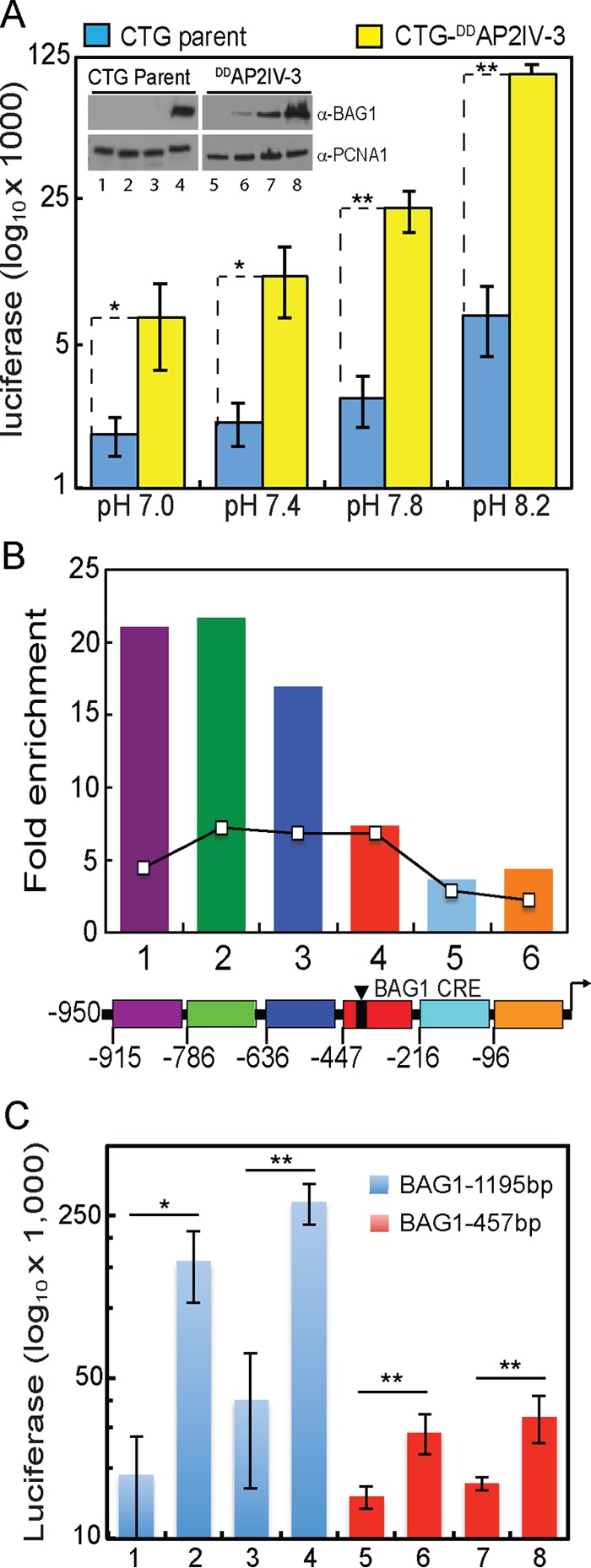
AP2IV-3 regulates BAG1 transcription. (A) Overexpression of AP2IV-3 induces the expression of the BAG1 promoter driving firefly luciferase (BAG1-Luc). CTG transgenic parasites expressing ^DD^AP2IV-3 and BAG1-Luc (yellow bars) and the CTG parent strain expressing BAG1-Luc alone (blue bars) were grown in medium as indicated (pH 7.0 to 8.2) plus Shield 1 (100 nM). Lysates from infected HFF cells were prepared and assayed for luciferase expression by standard methods. Average results from triplicate assays are graphed on a log_10_ scale because of the wide range of luciferase light unit values (note that no-extract background luciferase values were ~250 light units). The statistical significance of differences between matching CTG parent and CTG-^DD^AP2IV-3 luciferase results are indicated (*, *P* < 0.05; **, *P* < 0.01). (Inset) Western blot analysis run in parallel with the luciferase assays showing that Shield 1-induced overexpression of ^DD^AP2IV-3 elevated the expression of the native BAG1 protein as detected with an anti-BAG1 MAb. Lanes: 1 and 5, pH 7.0 medium; 2 and 6, pH 7.4 medium; 3 and 7, pH 7.8 medium; 4 and 8, pH 8.2 medium. *T. gondii* PCNA1 staining was included to demonstrate equal protein loading. (B) ^DD^AP2IV-3 occupies the native BAG1 promoter in parasite chromatin. Specific binding was monitored in specific sequences of the BAG1 promoter 5′ to the BAG1 coding region (−950 bp flanking, including the BAG1 5′ UTR up to the coding ATG). The region of the BAG1 promoter between −950 and −447 bp (purple, green, and blue regions) showed the highest enrichment and overlaps the previously identified regions where AP2IX-9 also binds, shown here as an overlaid reference line graph (6). Note that the BAG1 promoter element (BAG1 CRE) previously mapped as required for compound 1 induction (25) is located downstream of the regions with the highest AP2IV-3 binding. (C) Promoter deletion reveals that DNA sequences >457 bp upstream of the BAG1 ATG codon are required for maximum luciferase activity in ^DD^AP2IV-3 parasites. Luc expression constructs containing 1,195 bp (blue bars) or 457 bp (red bars) of the BAG1 promoter were introduced into the CTG-^DD^AP2IV-3 parasites, and CTG-^DD^AP2IV-3::BAG1(1,197 bp)-Luc and CTG-^DD^AP2IV-3::BAG1(457 bp)-Luc transgenic clones were isolated. Luciferase assays of parasites treated with various medium and Shield 1 conditions were performed on three independent clones for each promoter transgenic strain, and the average results were plotted on a log_10_ scale. Growth conditions: lanes 1 and 5, pH 7.0 medium; lanes 2 and 6, pH 7.0 medium plus 100 nM Shield 1; lanes 3 and 7, pH 8.2 medium; lanes 4 and 8, pH 8.2 medium plus Shield 1. The statistical significance of differences between Shield 1 plus versus minus luciferase results are indicated (*, *P* < 0.05; **, *P* < 0.01).

The global analysis of gene expression in AP2IV-3 transgenic strains demonstrates that deletion or overexpression of AP2IV-3 strongly affects canonical BAG1 expression in a manner consistent with AP2IV-3 functioning as a transcriptional activator. To confirm that AP2IV-3 directly regulates the BAG1 promoter, we generated a CTG transgenic strain coexpressing ^DD^AP2IV-3 and a second gene encoding firefly luciferase driven by the BAG1 promoter (BAG1-Luc). In previous studies, the BAG1 promoter conferred alkaline-stress-inducible luciferase expression on developmentally competent strains (25). As a control, we also isolated a CTG parental strain expressing the same BAG1-Luc reporter. Results from luciferase assays with 100 nM Shield 1 (Fig. 5A) demonstrated that luciferase expression was higher in the CTG-^DD^AP2IV-3::BAG1-Luc parasites (yellow bars) at all medium pHs (plus Shield 1) than in the CTG::BAG-Luc control strain (blue bars). This difference was strongly amplified by increasing alkaline stress; CTG-^DD^AP2IV-3::BAG1-Luc parasites cultured in pH 8.2 medium plus 100 nM Shield 1 expressed luciferase at ~20-fold higher levels than the CTG::BAG1-Luc control strain (Fig. 5A). The combination of increasing alkaline stress plus Shield 1 also elevated native BAG1 protein levels in a Shield 1-dependent manner over the CTG parent controls (Fig. 5A, inset Western blot assay). These results demonstrated that ^DD^AP2IV-3 was regulating not only the BAG1 promoter driving the Luc cassette but also the native BAG1 promoter.

The direct regulation of the BAG1 promoter by AP2IV-3 was further validated by chromatin immunoprecipitation (ChIP), followed by quantitative PCR (qPCR) (Fig. 5B). The binding of ^DD^AP2IV-3 to parasite genomic DNA demonstrated a specific preference for the BAG1 promoter. The pattern of ^DD^AP2IV-3 chromatin interaction overlaps the binding of AP2IX-9 to the BAG1 promoter published previously (6) and shown here as a reference (Fig. 5B, line graph). To control for nonspecific binding, we also analyzed ^DD^AP2IV-3 binding to the promoter of bradyzoite surface antigen SRS9, which is not regulated by AP2IV-3. No binding enrichment of ^DD^AP2IV-3 to the SRS9 promoter region was detected (data not shown). AP2IV-3 binding appeared to be stronger and to have a stronger preference for the −950 to −636 region than AP2IX-9 binding. To examine this binding preference further, we generated new stable CTG-^DD^AP2IV-3 transgenic clones expressing either the full-length BAG1 promoter (1,195 bp) or a truncated promoter (457 bp) missing the AP2IV-3 binding region as defined by the ChIP-qPCR experiments. The full-length and truncated BAG1-Luc reporters were both induced by alkaline stress or AP2IV-3 overexpression, which, when combined, gave the highest luciferase expression for each promoter construct (Fig. 5C). However, truncation of the BAG1 promoter substantially reduced overall luciferase activity, indicating that the loss of the region of active AP2IV-3 binding was required for full expression of the BAG1 promoter.

## DISCUSSION

**FIG 6:**
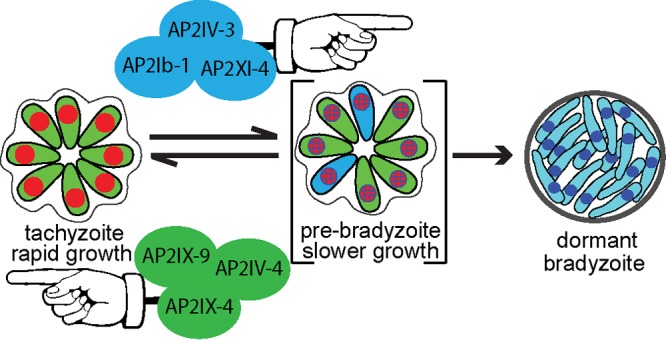
Model of ApiAP2 regulation of bradyzoite development.

We have confirmed here and previously (6) that two of the six *Toxoplasma* ApiAP2 factors (AP2IX-9 and AP2IV-3) induced early in bradyzoite development have important roles in the regulation of bradyzoite gene expression. AP2IX-9 is a transcriptional repressor that prevents the induction of bradyzoite gene expression and reduces tissue cyst formation (6), and thus, it was not surprising that disrupting AP2IX-9 in this study led to transgenic parasites that more readily formed tissue cysts under mild stress conditions. In contrast, AP2IV-3 is a transcriptional activator capable of enhancing tissue cyst formation when overexpressed or causing lower tissue cyst formation when deleted. Intriguingly, these two ApiAP2 factors regulate many of the same genes, and ChIP and luciferase promoter assays clearly demonstrated that AP2IX-9 and AP2IV-3 regulate the BAG1 promoter with opposite consequences and also specifically bind the BAG1 promoter in chromatin (Fig. 5B). We do not know if AP2IX-9 and AP2IV-3 directly compete or operate sequentially but separately to control bradyzoite gene expression. In addition to AP2IX-9 and AP2IV-3, there is evidence that two other ApiAP2 factors (AP2XI-4 and AP2IX-4) regulate BAG1 gene expression (27, 28). Therefore, at a minimum, BAG1 transcription is controlled by four ApiAP2 factors, two activators (AP2XI-4, AP2IV-3) and two repressors (AP2IX-9, AP2IX-4) (Fig. 6). Our discovery of multiple ApiAP2 factors regulating BAG1 is consistent with our earlier investigation (25) that revealed that the BAG1 promoter contains a number of potential repressor and activator promoter elements and that multiple nuclear protein complexes binding BAG1 promoter DNA fragments could be detected. Further studies are needed to precisely map the BAG1 promoter elements each factor binds and to determine whether these factors are contained in the same or different chromatin complexes.

Our group and others have established that bradyzoite development requires parasite replication (29) and also slowing of the tachyzoite cell cycle primarily by increasing the length of the G_1_ phase (30). The shift to slower growth in spontaneous models of development is quite rapid, occurring ~20 divisions following the emergence of the tachyzoite stage (30, 31). Importantly, early switching parasites have transcriptomes very similar to those of tachyzoites, compared to more mature bradyzoites (18), indicating that these early developing parasites are largely slowly growing tachyzoites that can be considered prebradyzoites (Fig. 6). ApiAP2 factors likely have important roles in the regulation of these growth-to-development transitions, and consistent with this idea, we have shown that the AP2IX-9 repressor promotes continued tachyzoite replication in type II and III strains while also preventing an increase in bradyzoite gene expression or production of tissue cysts (6). ApiAP2 factors may also influence the intravacuolar behavior of parasites. The cell cycle state and bradyzoite marker expression of individual parasites following an alkaline shift are asynchronous compared to the tightly synchronous cell cycles of tachyzoites replicating in the same vacuole (21). The molecular mechanisms responsible for intravacuole tachyzoite growth synchrony and bradyzoite developmental asynchrony are not understood. However, it is clear that ApiAP2 factor expression follows this dichotomy and may regulate these changes. Cyclical ApiAP2 factors in tachyzoites sharing the same vacuole are tightly coexpressed with specific ApiAP2 factor profiles providing markers for each cell cycle phase (12). In contrast, in alkali-stressed differentiating populations, ApiAP2 expression in parasites sharing the same vacuole is no longer tightly coordinated. By dual tagging of ApiAP2 factors in the same parasite, we have determined that AP2IX-9 is generally upregulated first, while peak expression of AP2IV-3 coincides with the decline in AP2IX-9 expression (Fig. 1B). However, these expression patterns were population based, not vacuole exclusive, as individual parasites within the same vacuole might express either or both nuclear factors. It is reasonable to suggest that it is likely to be more important for daughter tachyzoites to become infectious at the same time than for bradyzoites to become simultaneously dormant in the same tissue cyst, which may offer a biological rationale for the switch in ApiAP2 factor intravacuolar expression profile. There is also good biological logic to express an ApiAP2 bradyzoite repressor that promotes the tachyzoite stage earlier rather than later in the switching process, as we observed for AP2IX-9 (6). Importantly, the differential expression of ApiAP2 factors during bradyzoite development now offers an opportunity to better understand the transitions in this developmental pathway at the level of the individual parasite.

A growing body of data indicates that fulfilling the biotic requirement of the intermediate life cycle is largely the province of the tachyzoite stage and not that of parasites progressively engaged in developing and ultimately residing for the long term in the specialized tissue cyst. Oral infection with sporozoites or bradyzoites rapidly leads to population-wide redevelopment of the tachyzoite stage, which is responsible for parasitemia in animals (32, 33). A recent report demonstrates that the spread of tachyzoites into the vascular system in mice leads to tachyzoite infection of endothelial cells of the small capillaries of the brain that, upon lysis, travel across the blood-brain barrier to provide the primary parasite source of brain tissue cysts (34). Consistent with this discovery, a recent large study of *Toxoplasma* development in mice demonstrates that cyst size in brain tissue is related to the tachyzoite vacuole size at the time of switching (35), with the average cyst size and number becoming stable after the first few weeks postinfection (35, 36). Increases in tissue cyst size in later stages of chronic infection are largely independent of parasite replication (35), and there is little evidence that cycles of cyst rupture amplify tissue cyst numbers, as these events are rare and minimally productive (0.26% of brain cysts recrudesce) (37). Some replication of bradyzoites or prebradyzoites can be detected well into development (35), and there is evidence that tachyzoites formed from sporozoites are capable of switching to bradyzoites after very few divisions if artificially stressed (31). However, the contribution of mature bradyzoite replication to the biotic expansion of *Toxoplasma* in infected animals is likely very modest. Our recent discoveries of multiple ApiAP2 repressors and activators regulating growth and tissue cyst formation provide substantial support for the close relationship between the tachyzoite cell cycle and mechanisms that trigger the progressive development of the tissue cyst. In addition to the AP2IX-9 and AP2IV-3 factors studied here, our group recently identified two cell cycle-regulated ApiAP2 factors of the tachyzoite whose major functions are to repress bradyzoite gene expression (27, 28). Thus, there is now direct evidence of at least six ApiAP2 factors regulating bradyzoite development that act at the interface of tachyzoite growth and the stress responses that trigger the bradyzoite developmental pathway (model in Fig. 6). These results indicate that the competition between continued growth and the development of dormant tissue cysts required for transmission likely drove the evolution of developmental transcriptional mechanisms in *Toxoplasma*. Further, this model is consistent with our previous work indicating that bradyzoite differentiation is likely the default pathway of life cycle-competent strains (30, 31), and therefore, one role of ApiAP2 bradyzoite repressors is to ensure that the reproduction of tachyzoites meets minimum biotic requirements. Opposing transcriptional forces are a common adaptive feature of gene expression mechanisms from bacteria to humans (38) and are a particular theme of AP2 regulation of plant gene expression (39). Plant AP2 repressors serve specific roles in flower development (40) and the response to environmental stress (41), and there appear to be two kinds of AP2 repressors in plants, an active form that binds DNA directly and a type of repressor that operates by inhibiting activator activity (39). Our studies of ApiAP2 factors may have uncovered both types of repressors in *Toxoplasma* (6, 27).

It is early in our understanding of how ApiAP2 factors function in *Toxoplasma* and other apicomplexans, although there is already more complexity in coccidian ApiAP2 mechanisms than what might have been expected. Coccidian parasites have more ApiAP2 factors than other apicomplexan parasite families, such as the *Plasmodium* spp*.*, which can partially be explained by a larger genome and transcriptome. Another possible explanation is the wider intermediate host ranges of coccidian parasites, and this may explain the relatively few ApiAP2 factors exclusively expressed in the feline definitive life cycle (Table 1). In other eukaryotes, including mammals, cell division is a requirement for subsequent development, as the process of chromosome replication allows for a specialized transcriptional programing of terminally differentiated cells (42, 43). Intriguingly, *Toxoplasma* appears to be able to tap into these mechanisms to find long-lived host tissue to develop the tissue cyst (44). It will be interesting to determine whether host-parasite signal transduction mechanisms acting through ApiAP2 factor mechanisms are responsible for achieving the coordination of tissue cyst formation in terminally differentiated host cells. There are clearly candidates for host factors involved in this communication pathway that negatively regulate host cell growth, such as CDA-1, p21, and cyclin B1 (1, 44, 45), and *Toxoplasma* kinases that are likely involved, such as casein kinase (1) and *Toxoplasma gondii* protein kinase A (46). Therefore, ApiAP2 factors are intriguing candidates that could receive critical signals from these and other host pathways and drive the appropriate developmental transcriptional response within the parasite.

## MATERIALS AND METHODS

### Cell culture and transgenic strains.

Parasite cultures were maintained in primary human foreskin fibroblasts (HFF) as previously described (47). All of the genetically modified strains generated here (for the full list of parent and transgenic strains, see Data Set S3) were grown in confluent monolayers of HFF cells in Dulbecco’s modified Eagle medium with 4.5 g/liter glucose, l-glutamine, and sodium pyruvate (Corning Cellgro); 5% fetal bovine serum (Sigma); and 1% penicillin-streptomycin-amphotericin B (HyClone) at 37°C with 5% CO_2_.

10.1128/mSphere.00347-16.5 DATA SET S3  Transgenic strains produced in this study and key primer sequences. Download DATA SET S3, XLSX file, 0.01 MB. Copyright © 2017 Hong et al. 2017 Hong et al. This content is distributed under the terms of the Creative Commons Attribution 4.0 International license.

### Overexpression of AP2IV-3.

For conditional overexpression, the AP2IV-3 single exon coding region was PCR amplified from genomic DNA to incorporate in-frame Xma1/Sbf1 sites, which were used to clone the purified PCR fragment into the pCTDDHA3x plasmid. This cloning results in the fusion of the FKBP peptide (11.2 kDa) and three copies of the HA epitope for detection (4.4 kDa) in frame with the N terminus of the AP2IV-3 protein with a final fusion protein mass of ~162 kDa (designated ^DD^AP2IV-3). The plasmid pCTDDHA3x-AP2IV-3 was introduced by electroporation into the type III CTG strain. Transgenic parasites were selected with chloramphenicol (20 μM), and clones were isolated by limiting dilution. Overexpression of AP2IV-3 was achieved by adding 100 nM Shield 1 synthesized in house to the culture medium.

### AP2IV-3 and AP2IX-9 knockout and complementation.

For disruption of ApiAP2 factors, we used the multi-guide-RNA (multi-gRNA) CRISPR-Cas9 system. The gRNA for each gene was generated by the CRISPR gRNA design tool supported by DNA2.0 (ATUM, Newark, CA). The CRISPR-Cas9 gRNA plasmid (pSAG1::Cas9-U6::sgUPRT) was provided by David Sibley (Washington University, St. Louis, MO). The single gRNA in this plasmid was replaced with a short oligonucleotide matching each targeted gene (see Data Set S3) by Q5 DNA site-directed mutagenesis (NEB, Ipswich, MA) with a common reverse primer (5′AACTTGACATCCCCATTTAC3′). Gene-specific ApiAP2 CRISPR-Cas9-gRNA plasmids (see Data Set S3) were sequenced to confirm the correct sequence of the gRNA. To generate clean knockouts in *Toxoplasma* tachyzoites, two gRNA plasmids for AP2IV-3 and four gRNA plasmids for AP2IX-9 were designed in the same gene. A plasmid named 3Frag-AP2KO.DHFR-TS containing a pyrimethamine resistance cassette was constructed by 3-fragment Gateway protocols (Thermo Fisher Scientific, Waltham, MA). Briefly, both the 5′ and 3′ untranslated regions (UTRs) of ApiAP2 genomic coding regions were PCR amplified from type I genomic DNA and used to create Gateway entry clones. The 5′ UTR was cloned into the pDONR_P4-P1r vector, and the 3′ UTR was cloned into the pDONR_P2r-P3 vector, by the BP recombination reaction. After isolation of the entry clones, pDONR_P4-P1r_AP2 and pDONR_P2r-P3_AP2 were combined in an LR recombination reaction with the p221-DHFR and pDEST_R4-R3 vectors to create the pDEST_AP2_DHFR knockout plasmid. The final plasmids were introduced into Pru and CTG strains, and transgenic parasites were selected by using pyrimethamine, followed by clonal isolation by limiting dilution. Thirty micrograms each of the gRNA plasmid and the pDEST_AP2_DHFR knockout plasmid was transfected into 3 × 10^7^ parasites by electroporation. For complementation of AP2IV-3, purified cosmid PSBLJ40 carrying a single copy of the AP2IV-3 gene was transfected into Pru-Δ*ap2IV-3* parasites. To quantify genetic rescue, established drug-resistant populations were tested for tissue cyst formation in alkaline medium (pH 8.2) and AP2IV-3 mRNA and control glyceraldehyde-3-phosphate dehydrogenase (GAPDH) mRNA were analyzed by reverse transcription (RT)-qPCR.

### Immunofluorescence assay and Western analysis.

Parasites were inoculated onto confluent HFF coverslips and incubated for the times indicated, and IFA was performed with the following primary reagents: anti-HA antibody (Roche rat monoclonal antibody [MAb] 3F10, 1:500), anti-myc antibody (Santa Cruz Biotechnology mouse MAb, 1:500), biotin-labeled DBA (1:3,000; Vector Labs, CA), and 4',6-diamidino-2-phenylindole (DAPI; 0.5 mg/ml). All Alexa (Molecular Probes, CA)- and streptavidin (Vector Labs, CA)-conjugated secondary antibodies were used at 1:1,000. Image acquisition was performed with a Zeiss Axiovert microscope equipped with a 100× objective. Western blotting with a specific antibody monitored the protein expression in *Toxoplasma* parasites. Purified parasites were lysed in SDS-PAGE sample buffer with Laemmli loading dye, heated at 95°C for 10 min, and briefly sonicated. After separation on the gel, proteins were transferred onto nitrocellulose membrane, detected with MAbs and horseradish peroxidase-conjugated secondary antibodies (Jackson ImmunoResearch, PA), and visualized by enhanced-chemiluminescence detection (PerkinElmer).

### Microarray analysis.

Total RNA was purified from intracellular CTG, CTG-^DD^AP2IV-3, and CTG-Δ*ap2IV-3* parasites maintained in pH 7.0 and 7.8 media (32 to 36 h postinfection) or following 48 h of exposure to pH 8.2 medium with the RNeasy minikit (Qiagen) according to the manufacturer’s instructions. Two biological replicates were prepared for each parasite line under each experimental condition, and RNA quality was assessed with the Agilent Bioanalyzer 2100 (Agilent Technology). Following fragmentation, 10 μg of cRNA was hybridized for 16 h at 45°C to *Toxoplasma* Affymetrix U133 Plus 2.0 arrays. GeneChips were washed and stained in the Affymetrix Fluidics Station 450. Hybridization data were analyzed with the GeneSpring GX software package (v12.6.1; Agilent).

### Luciferase assays.

Luciferase assays were performed according to manufacturer’s protocols (Promega, Madison, WI) with modifications as previously described (25). Briefly, HFF cells in T25 25-cm^2^ flasks were inoculated with CTG-BAG1-Luc and CTG-^DD^AP2IV-3::BAG1-Luc transgenic parasites at a multiplicity of infection (MOI) of 3:1, parasites were allowed to invade cells for 1 h, and the culture medium was changed to either pH 8.2 or pH 7.0 medium plus or minus 100 nM Shield 1. The alkaline-stress-shifted cultures were grown under non-CO_2_ conditions for 48 h and then harvested for whole-cell lysates according to the manufacturer’s protocols. Each experimental condition was assessed in three independent cultures.

### ChIP and qPCR.

Parasites were inoculated at an MOI of 3:1 into T175 175-cm^2^ flasks, allowed to invade cells for 1 h, and rinsed three times with Hanks balanced salt solution to remove free-floating parasites, and the medium was replaced with pH 8.2 medium supplemented with 100 nM Shield 1. ChIP and qPCR were performed by previously published methods (6).

### Accession number(s).

All of the microarray data from this study have been deposited at NCBI GEO under accession no. GSE89469.
