# Supplementary material for: Opposing Transcriptional Mechanisms Regulate Toxoplasma Development
Source: mSphere. 2017 Feb 22;2(1):e00347-16. doi: 10.1128/mSphere.00347-16 (PMC5322347; doi:10.1128/mSphere.00347-16)
Supplement: TABLE S1 [file sph001172236st1.docx]

**Table S1. ApiAP2 mRNA expression in Type II strains across *Toxoplasma* life cycle stages.**

**mRNA expression analysis**. Microarray data: ME49 strain tachyzoite and 2 day *in vitro* bradyzoite. RNA-seq data: M4 strain 21 day *in vivo* bradyzoite,10 day oocyst (sporozoites), CZ-H3 strain tachyzoite and 3 day post-infection of cats (merozoites). All 67 *Toxoplasma* ApiAP2 factors were ranked from highest (rank 1) to lowest (rank 67) level of expression in each sample. The percentile expression indicated in parentheses is included to provide a reference for the relative expression of each ApiAP2 mRNA against all genes expressed in the RNA sample. **The highest levels of mRNA expression for developmental ApiAP2s are indicate by larger bold font.** Column color code: yellow=in vitro tachyzoites, green=bradyzoites, red=fully sporulated oocysts containing sporozoites, blue=merozoites, orange=tachyzoite cell cycle regulated ApiAP2s with the highest amplitude ApiAP2 mRNAs indicated as *dynamic cell cycle*. Note: ApiAP2 mRNAs that are transiently expressed are not fully captured particularly dynamic cell cycle factors (e.g. AP2IV-4 75th percentile only S phase-mitosis). For some ApiAP2 factors, mRNA expression has been confirmed at the protein level indicated by (P). Strain-specific differences in expression are evident in some data but were not highlighted.

| **AP2 name**  **cell cycle**  **orange** | **ME49 tachy** | **ME49**  **2d brady** | **M4**  **21d brady** | **M4-10d oocyst** | **CZ-H3 tachy** | **CZ-H3**  **3d mero** | **Developmental Expression**  *see additional evidence ToxoDB  (P) confirmed at the protein level |
| --- | --- | --- | --- | --- | --- | --- | --- |
| **AP2Ib-1** | 62^(11)^ | **28^(60)^** | 57^(22)^ | 37^(33)^ | 63^(18)^ | **21^(53)^** | early bradyzoites (P)  & feline cycle stages* |
| **AP2III-1** | 56^(25)^ | 64^(15)^ | 65^(5)^ | 52^(21)^ | 65^(8)^ | 62^(21)^ | no clear stage pattern |
| **AP2III-2** | **9^(72)^** | **22^(62)^** | **24^(66)^** | **16^(64)^** | **25^(62)^** | 63^(20)^ | intermediate life cycle (P)  & highest in unsporulated oocysts* |
| **AP2III-3** | 67^(1)^ | 67^(0)^ | 66^(5)^ | **13^(70)^** | 67^(6)^ | 65^(15)^ | sporozoite specific |
| **AP2III-4** | 66^(1)^ | 65^(4)^ | 63^(11)^ | 31^(41)^ | 64^(15)^ | **17^(58)^** | feline cycle specific |
| **AP2IV-1** | 37^(47)^ | 30^(53)^ | 40^(38)^ | **11^(72)^** | 28^(55)^ | **10^(68)^** | highest in feline cycle stages*  & oocyst sporulation* |
| **AP2IV-2^+^** | 34^(48)^ | 29^(54)^ | 36^(42)^ | 45^(27)^ | 59^(20)^ | 26^(52)^ | no clear stage pattern |
| **AP2IV-3** | 64^(9)^ | **13^(72)^** | 33^(48)^ | **6^(91)^** | 58^(21)^ | **7^(71)^** | early bradyzoites (P),  sporozoites & merozoites |
| **AP2IV-4** | 33^(49)^ | 52^(37)^ | 27^(61)^ | 28^(46)^ | 31^(50)^ | 29^(49)^ | likely tachyzoite-specific  *dynamic cell cycle* (P) |
| **AP2IV-5** | **21^(62)^** | 49^(38)^ | 58^(19)^ | **10^(73)^** | **15^(74)^** | 44^(33)^ | tachyzoites & sporozoites |
| **AP2V-1** | **24^(59)^** | 37^(47)^ | 57^(22)^ | 27^(48)^ | **13^(77^**^)^ | 59^(20)^ | highest in tachyzoites |
| **AP2V-2** | 60^(21)^ | 63^(16)^ | 65^(5)^ | 24^(49)^ | 45^(37)^ | 54^(24)^ | no clear stage pattern |
| **AP2VI-1^+^** | **2^(89)^** | **3^(87)^** | **2^(95)^** | **4^(93)^** | **6^(86)^** | **2^(78)^** | high expression in all stages  *dynamic cell cycle* (P) |
| **AP2VI-2** | 54^(29)^ | 32^(50)^ | 34^(46)^ | **18^(59)^** | 32^(50)^ | 33^(44)^ | highest in unsporulated oocysts* |
| **AP2VI-3** | 52^(30)^ | **35^(49)^** | 55^(25)^ | 44^(27)^ | 49^(35)^ | **5^(72)^** | highly expressed in merozoites & possibly early bradyzoites |
| **AP2VIIa-1** | 55^(29)^ | **24^(61)^** | **20^(73)^** | **3^(93)^** | 52^(30)^ | 32^(45)^ | bradyzoites & sporozoites *dynamic cell cycle* (P) |
| **AP2VIIa-2** | **1^(92)^** | **2^(89)^** | **1^(97)^** | 39^(32)^ | **3^(90)^** | 40^(38)^ | intermediate life cycle (P)  & unsporulated oocysts* |
| **AP2VIIa-3** | **3^(88)^** | **5^(85)^** | **11^(85)^** | **2^(95)^** | **5^(86)^** | 36^(40)^ | intermediate life cycle (P)  & sporozoites |
| **AP2VIIa-4** | **19^(63)^** | **8^(83)^** | **21^(71)^** | 33^(38)^ | **23^(64)^** | **16^(59)^** | intermediate life cycle (P)  & merozoites |
| **AP2VIIa-5** | **11^(70)^** | **25^(61)^** | 39^(39)^ | 65^(06)^ | **29^(54)^** | 34^(43)^ | highest in tachyzoites (P)  & early bradyzoites |
| **AP2VIIa-6** | **14^(68)^** | **17^(67)^** | 35^(45)^ | **8^(86)^** | 47^(37)^ | 64^(20)^ | highest in sporozoites also moderate in tachyzoites (P)/early bradyzoites |
| **AP2VIIa-7** | 35^(48)^ | 44^(44)^ | 31^(49)^ | 23^(51)^ | 27^(58)^ | 51^(28)^ | 86th percentile in unsporulated oocysts* |
| **AP2VIIa-8** | **27^(54)^** | **18^(65)^** | **13^(81)^** | **5^(93)^** | **19^(67)^** | 66^(12)^ | intermediate life cycle & sporozoites, *dynamic cell cycle* (P) |
| **AP2VIIa-9** | 51^(30)^ | 43^(45)^ | **18^(74)^** | 66^(06)^ | 37^(47)^ | **13^(62)^** | mature bradyzoites? &  feline life cycle* |
| **AP2VIIb-1** | 40^(43)^ | 36^(48)^ | 43^(37)^ | 22^(52)^ | 39^(42)^ | 41^(30)^ | no clear stage pattern |
| **AP2VIIb-2** | 53^(29)^ | 50^(37)^ | 41^(38)^ | 17^(60)^ | 44^(37)^ | 22^(52)^ | no clear stage pattern |
| **AP2VIIb-3** | **12^(69)^** | **9^(81)^** | **8^(86)^** | 42^(27)^ | **22^(64)^** | 42^(37)^ | intermediate life cycle (P) |
| **AP2VIII-1** | 39^(44)^ | 48^(38)^ | 50^(30)^ | 58^(16)^ | 36^(46)^ | 48^(30)^ | no clear stage pattern |
| **AP2VIII-2** | 41^(43)^ | 54^(36)^ | 38^(40)^ | 49^(24)^ | 46^(36)^ | 47^(30)^ | no clear stage pattern |
| **AP2VIII-3** | **10^(71)^** | **14^(70)^** | **19^(73)^** | **12^(70)^** | **21^(64)^** | 18^(56)^ | intermediate life cycle  & sporozoites |
| **AP2VIII-4** | 36^(48)^ | **26^(60)^** | **30^(52)^** | 60^(16)^ | 48^(36)^ | 58^(23)^ | possible bradyzoite specific* |
| **AP2VIII-5** | **16^(66)^** | **15^(69)^** | 32^(48)^ | 21^(52)^ | **20^(66)^** | **1^(82)^** | tachyzoites, early bradyzoites & merozoites |
| **AP2VIII-6** | **8^(75)^** | **6^(85)^** | **7^(91)^** | 63^(12)^ | **7^(85)^** | **14^(61)^** | intermediate life cycle & merozoites |
| **AP2VIII-7** | **4^(88)^** | **1^(91)^** | **4^(94)^** | 34^(37)^ | **1^(92)^** | **15^(61)^** | intermediate cycle (P) & merozoites |
| **AP2IX-1** | 63^(10)^ | 60^(25)^ | 52^(29)^ | **15^(66)^** | 54^(25)^ | **6^(74)^** | feline cycle stages* & sporozoites |
| **AP2IX-2^+^** | 50^(32)^ | 42^(46)^ | N/A | N/A | 60^(19)^ | 57^(23)^ | no clear stage pattern |
| **AP2IX-3** | 44^(40)^ | 46^(39)^ | 62^(12)^ | 19^(59)^ | 61^(19)^ | 62^(20)^ | no clear stage pattern |
| **AP2IX-4** | **7^(76)^** | **4^(87)^** | **3^(95)^** | 38^(32)^ | **26^(59)^** | 49^(29)^ | intermediate life cycle  *dynamic cell cycle* (P) |
| **AP2IX-5** | 28^(52)^ | 41^(47)^ | 48^(31)^ | 43^(27)^ | 18^(66)^ | 43^(36)^ | no clear stage pattern |
| **AP2IX-6** | 43^(42)^ | 33^(49)^ | 46^(32)^ | 40^(30)^ | 38^(44)^ | **8^(70)^** | feline cycle stages* |
| **AP2IX-7** | 57^(25)^ | 61^(23)^ | 56^(25)^ | 61^(15)^ | 53^(28)^ | 53^(25)^ | no clear stage pattern |
| **AP2IX-8** | **17^(66)^** | **23^(62)^** | **25^(66)^** | 56^(19)^ | **16^(72)^** | **20^(52)^** | intermediate life cycle & possibly merozoites |
| **AP2IX-9** | 58^(24)^ | **12^(72)^** | **5^(93)^** | 41^(29)^ | 50^(33)^ | 25^(52)^ | early bradyzoite specific, protein was not detected *in vivo* cysts (P) |
| **AP2X-1** | 47^(37)^ | 58^(28)^ | 45^(34)^ | 59^(16)^ | 40^(42)^ | 38^(39)^ | no clear stage pattern |
| **AP2X-2^+^** | 30^(50)^ | 27^(60)^ | 49^(30)^ | **7^(90)^** | 41^(40)^ | 24^(52)^ | sporozoite specific |
| **AP2X-3^+^** | 42^(42)^ | 38^(47)^ | 47^(31)^ | 54^(20)^ | 55^(24)^ | 23^(52)^ | no clear stage pattern |
| **AP2X-4** | **13^(69)^** | **21^(64)^** | **28^(60)^** | 57^(20)^ | **17^(69)^** | **11^(67)^** | intermediate cycle & feline cycle stages |
| **AP2X-5** | **23^(60)^** | 56^(32)^ | **9^(86)^** | **14^(66)^** | **10^(80)^** | 35^(41)^ | tachyzoites (P), mature bradyzoites & sporozoites |
| **AP2X-6** | 65^(4)^ | 66^(4)^ | 60^(17)^ | 29^(44)^ | 57^(22)^ | 46^(31)^ | no clear stage pattern |
| **AP2X-7** | **5^(86)^** | **7^(84)^** | **10^(86)^** | **9^(90)^** | **8^(85)^** | 19^(55)^ | intermediate life cycle & sporozoites |
| **AP2X-8** | 46^(39)^ | 55^(34)^ | 44^(36)^ | 36^(34)^ | 51^(32)^ | 60^(23)^ | no clear stage pattern (P) |
| **AP2X-9** | **6^(82)^** | **11^(73)^** | **22^(68)^** | 32^(39)^ | **2^(91)^** | **12^(63)^** | intermediate life cycle (P) & merozoites* |
| **AP2X-10** | 59^(24)^ | 47^(38)^ | 54^(25)^ | **1^(96)^** | 62^(18)^ | **28^(49)^** | sporozoites & feline cycle* |
| **AP2X-11** | 45^(39)^ | 51^(37)^ | 42^(37)^ | 47^(25)^ | 34^(48)^ | 39^(39)^ | no clear stage pattern |
| **AP2XI-1** | 20^(63)^ | 10^(74)^ | 61^(16)^ | 26^(48)^ | 56^(24)^ | 56^(24)^ | no clear stage pattern  *dynamic cell cycle* (P) |
| **AP2XI-2** | **26^(56)^** | **16^(67)^** | **15^(77)^** | 62^(14)^ | **14^(74)^** | 30^(47)^ | intermediate life cycle (P) |
| **AP2XI-3** | 29^(50)^ | 45^(43)^ | 26^(62)^ | 51^(22)^ | 12^(77)^ | 4^(76)^ | no clear stage pattern  *important G1 regulator* (P) |
| **AP2XI-4** | **18^(66)^** | **20^(64)^** | **29^(53)^** | 46^(25)^ | 43^(39)^ | 52^(27)^ | likely intermediate cycle specific  *dynamic cell cycle* (P) |
| **AP2XI-5** | 25^(57)^ | 31^(53)^ | 14^(79)^ | 30^(43)^ | 9^(81)^ | 3^(77)^ | no clear stage pattern (P) |
| **AP2XII-1** | 31^(50)^ | 39^(47)^ | 6^(92)^ | 50^(23)^ | 4^(86)^ | 55^(23)^ | no clear stage pattern |
| **AP2XII-2** | 38^(44)^ | 53^(37)^ | 51^(30)^ | 42^(28)^ | 35^(47)^ | 37^(40)^ | no clear stage pattern  *dynamic cell cycle* |
| **AP2XII-3** | 61^(20)^ | 62^(18)^ | 64^(11)^ | 35^(36)^ | 66^(6)^ | 67^(4)^ | unsporulated oocyst specific* |
| **AP2XII-4** | 15^(67)^ | 34^(49)^ | 23^(67)^ | 25^(49)^ | 33^(49)^ | 31^(46)^ | no clear stage pattern (P) |
| **AP2XII-5** | 48^(35)^ | 57^(30)^ | 17^(76)^ | 20^(53)^ | 42^(39)^ | 45^(33)^ | no clear stage pattern (P) |
| **AP2XII-6** | **32^(50)^** | **19^(64)^** | 59^(17)^ | 55^(20)^ | **30^(51)^** | **9^(69)^** | tachyzoites and early bradyzoites  & feline cycle |
| **AP2XII-8** | **22^(61)^** | 40^(47)^ | **16^(76)^** | 48^(25)^ | **11^(78)^** | 27^(50)^ | likely intermediate cycle specific* |
| **AP2XII-9** | 49^(33)^ | 59^(26)^ | 37^(41)^ | 53^(20)^ | 24^(62)^ | 50^(29)^ | no clear stage pattern  *dynamic cell cycle* (P) |
|  | 11 | 15 | 21 | 13 | 16 | 8 | # AP2s ≥ 70% per sample |
|  | 37 | 36 | 36 | 43 | 36 | 41 | # AP2s ≤ 50% per sample |
| Other data comments | | | - # of ApiAP2 factors expressed at <60 percentile in all samples=18 ApiAP2 factors - A single ApiAP2 factor is highly expressed in all samples, AP2VI-1 | | | | |
| Data sources | | | - all normalized and percentile values were obtained from ToxoDB - ^+^data for 5 ApiAP2 factors missing from ToxoDB was recovered from original data - *Other ToxoDB data used in this analysis; acute vs 28 day CBA/J infected brain tissue (RNA-seq), 29 *Toxoplasma* strains (RNA-seq), RH synchronized tachyzoite cell cycle mRNAs (microarray) | | | | |
| Protein confirmation sources | | | - White, Sullivan, Kim, unpublished; AP2Ib-1, AP2III-2, AP2IV-3 (this paper), AP2IV-4, AP2VIIa-5, AP2VIIa-6, AP2VIIb-3, AP2VIII-7, AP2IX-4, AP2XII-4, AP2XII-5 - reference 6: AP2IX-9, AP2VI-1; reference 10: AP2X-5, AP2X-8, AP2X-9; reference 12: AP2VIIa-1, AP2VIIa-4, AP2VI-1, AP2XI-1, AP2XII-9; reference 26: AP2XI-4 | | | | |
