# Supplementary figures and images for: Opposing Transcriptional Mechanisms Regulate Toxoplasma Development
Source: mSphere. 2017 Feb 22;2(1):e00347-16. doi: 10.1128/mSphere.00347-16 (PMC5322347; doi:10.1128/mSphere.00347-16)

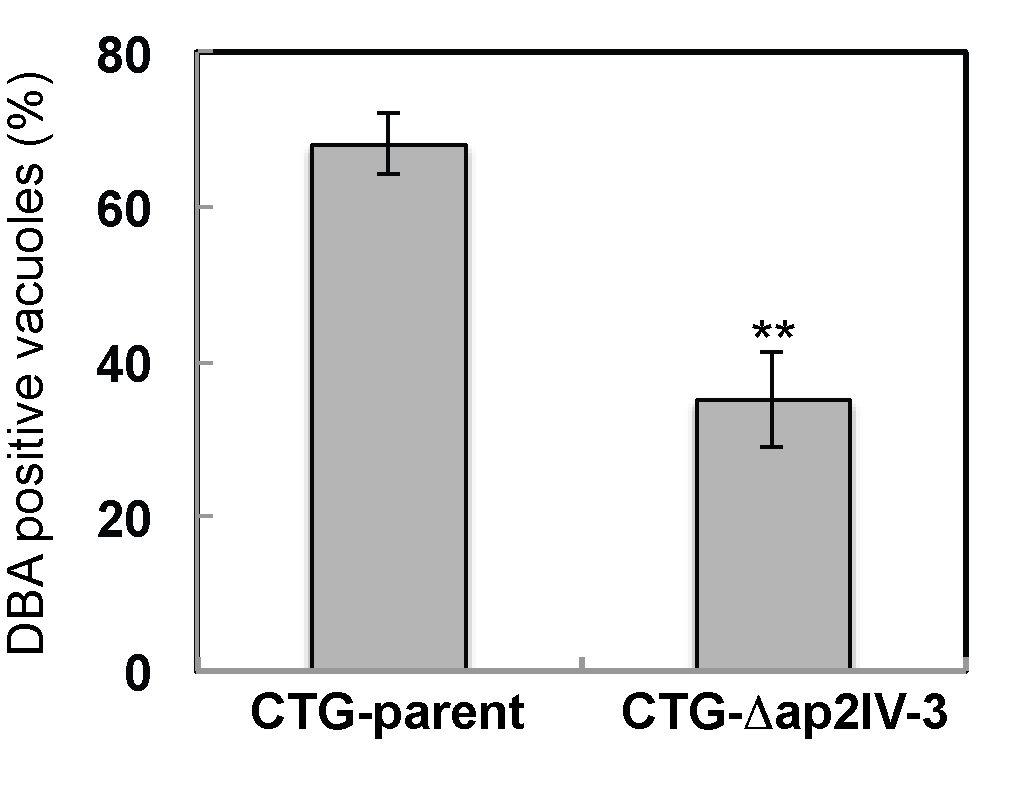

Supplement: FIG S1 [file sph001172236sf2.tif]
